# Beyond the Maximum Storage Capacity Limit in Hopfield Recurrent Neural Networks

**DOI:** 10.3390/e21080726

**Published:** 2019-07-25

**Authors:** Giorgio Gosti, Viola Folli, Marco Leonetti, Giancarlo Ruocco

**Affiliations:** 1 Center for Life Nanoscience, Istituto Italiano di Tecnologia, Viale Regina Elena 291, 00161 Rome, Italy; 2 CNR NANOTEC-Institute of Nanotechnology c/o Campus Ecotekne, University of Salento, Via Monteroni, 73100 Lecce, Italy; 3 Department of Physics, Sapienza University of Rome, Piazzale Aldo Moro 5, 00185 Rome, Italy

**Keywords:** recurrent neural networks, Hopfield neural networks, pattern storage

## Abstract

In a neural network, an autapse is a particular kind of synapse that links a neuron onto itself. Autapses are almost always not allowed neither in artificial nor in biological neural networks. Moreover, redundant or similar stored states tend to interact destructively. This paper shows how autapses together with stable state redundancy can improve the storage capacity of a recurrent neural network. Recent research shows how, in an *N*-node Hopfield neural network with autapses, the number of stored patterns (*P*) is not limited to the well known bound 0.14N, as it is for networks without autapses. More precisely, it describes how, as the number of stored patterns increases well over the 0.14N threshold, for *P* much greater than *N*, the retrieval error asymptotically approaches a value below the unit. Consequently, the reduction of retrieval errors allows a number of stored memories, which largely exceeds what was previously considered possible. Unfortunately, soon after, new results showed that, in the thermodynamic limit, given a network with autapses in this high-storage regime, the basin of attraction of the stored memories shrinks to a single state. This means that, for each stable state associated with a stored memory, even a single bit error in the initial pattern would lead the system to a stationary state associated with a different memory state. This thus limits the potential use of this kind of Hopfield network as an associative memory. This paper presents a strategy to overcome this limitation by improving the error correcting characteristics of the Hopfield neural network. The proposed strategy allows us to form what we call an absorbing-neighborhood of state surrounding each stored memory. An absorbing-neighborhood is a set defined by a Hamming distance surrounding a network state, which is an absorbing because, in the long-time limit, states inside it are absorbed by stable states in the set. We show that this strategy allows the network to store an exponential number of memory patterns, each surrounded with an absorbing-neighborhood with an exponentially growing size.

## 1. Introduction

Hopfield neural networks are a particular kind of recurrent neural networks (RNN), for this reason, we may refer to them as Hopfield RNN. Hopfield RNN have several applications, [1,2]. Among others, these neural networks can be used as storage devices capable of storing patterns, and can model “associative memory”. The general concept in this applications is that the attractor states of these neural networks can be considered as stored patterns. This is possible because, given sufficient time, Hopfield neural networks assign to each initial activation pattern $\bar{\sigma_{i}}$ a final activation pattern which can be composed by a steady state $\bar{\sigma_{o}}$, or a limit cycle composed of several states. Consequently, if we set the values of the initial activation pattern $\bar{\sigma_{i}}$ to an input vector, which we call stimulus, we find that the network evolves from $\bar{\sigma_{i}}$ to a final activation pattern that corresponds to a recovered memory pattern. Thus, in this framework, an RNN is a dynamical system, which connects an input-stimulus to an output-memory, and can be regarded as a content addressing memory that connects input states to output states.

This is possible because a Hopfield neural network is an RNN defined in discrete-time, and it is a deterministic dynamical system. This is why a Hopfield RNN is completely characterized by *N* neurons connected in a network formed of at most $N^{2}$ edges, the weights of which form the connectivity matrix J. The neural network’s instantaneous state is defined by a neuron activation vector $\bar{\sigma }$, which in Hopfield RNNs is a binary vector. The collection of all the neurons’ activation vectors $\bar{\sigma }$ forms a discrete space that contains all the possible $2^{N}$ binary vectors. The neural activation vectors $\bar{\sigma }$ may be separated in three categories: states that belong to limit cycles, steady states, and transient states. Limit cycles are neural activation states that repeat cyclically after a given number of transitions. Steady states are states that do not change in time. Transient states are neither limit cycles nor steady states, thus they are states that, if visited, are never visited again. Clearly, steady states may be regarded as limit cycles of length 1. Because a Hopfield RNN has a deterministic dynamics, any state $\bar{\sigma }\left(t\right)$ can only transit into a single state $\bar{\sigma }\left(t+1\right)$. Consequently, any input pattern always converges to the same limit cycle or steady state. For this reason, RNNs do not exclusively model associative memories, but more generally RNNs model how responses are associated with stimuli in stimulus-response behaviors.

It has been shown that, if we train an RNN with Hebbian learning, in the long time limit, all the network’s limit cycle are steady states, while, more generally, deterministic RNN may evolve to limit cycles of length larger than 1. In conclusion, Hopfield RNNs may have interesting applications as memory storage devices because, in principle, a Hopfield RNN can store an arbitrary number of $2^{N}$ binary vectors in a structure defined by $N^{2}$ continuous parameters [3,4,5].

RNNs belong to a broader class of synchronous distributed systems that perform tasks collectively [6,7]. Because RNNs offer a versatile model of stimulus response association, RNN models are not restricted to memory storage. Indeed, neuroscientists started using RNNs to model brain activity in different cognitive tasks. Monte et al. [8] and Carnevale et al. [9] use RNNs respectively to model the prefrontal cortex integration of context information, and the response modulation of the premotor cortex. The majority of approaches that use RNNs to model the storage of responses in association with stimuli can be divided into two main categories, the “Hebbian” approach and the innate approach. The “Hebbian” approach was proposed by Hebb himself [8]. In this approach, the neural network starts without connections between neurons, and, at each time, a new memory pattern is stored in the neural network, connections are added or updated to allow the new memory pattern to become a stationary state in the network dynamics. Consequently, in these systems, we have a recall when a state is absorbed by the correctly associated memory. Moreover, we get a recall error when a previously stored memory fails to form a stable state. Thus, when the network activation states are set to the memory, the network evolves outside of the memory. A limit of Hebb’s approach is that, as new patterns are stored, they start interfering with the previously stored patterns. The second approach is the “innate” approach [5,10], in which the neural network is innate and does not change. New memories are added, by associating to each new memory an existing stable state through an addressing system. Perin et al. find that innate neural assemblies can be found in the rats’ neural cortex [10]. Moreover, they present data that shows how analogous neural assemblies from different animals have similar connectivity properties, and they argue that these assemblies are used as building blocks for the formation of composite complex memories. In both learning strategies, the neural network J is represented by a set of interconnected neurons with an associated RNN model dynamics. The major difference between the two approaches is how J develops. For either learning strategy, the limit of the number of stimuli-response associations that can be stored in a neural network J is given by the maximum storage capacity *C* which is bounded by the number of attractors in the RNN. In this paper, we focus on a generalized form of the “Hebbian” approach in which we allow autapses and we surround each stored memory with a neighborhood of redundant stable states. This approach allows us to greatly overcome the well known storage limit of the more traditional Hopfield RNN constructed with a Hebbian approach.

The storage capacity limit of Hopfield RNNs without autapses was immediately recognized by Amit, Gutfreund, and Sompolinsky [11,12]. This limit is linear with *N* because the attempt to store a number *P* of memory elements larger than $\alpha_{c}P$, with $\alpha_{c}\approx 0.14$, results in a “divergent” number of retrieval errors (order *P*). In plain words, for a Hopfield RNN to be effective, the retrieval error probability must be low, and a Hopfield RNN can only be efficient if the stored memories do not exceed 14% of the network size. This strongly limits the application of neural networks for information storage. Nevertheless, this observation is hard to reconcile with what we observe in randomly generated (symmetric) neural networks, where the number of limit behaviours is exponentially large $2^{\gamma N}$ with γ=0.29 [13,14], which leads us to believe the opposite conclusions.

After Amit et al. [11,12] results, the following research focused on how modifications of the Hebbian rule affected the behavior of a Hopfield RNN, with the objective of finding more efficient learning strategies. Abu-Mostafa and St. Jaques [15] claimed that, for a Hopfield model with a generic coupling matrix, the upper bound value of the storable patterns is the number of neurons *N* (i.e., P<N). Immediately afterwards, McEliece and colleagues [16], found an even more severe upper bound when considering matrices constructed exclusively with the Hebbian dyadic form. The authors in [16] claims that the maximum *P* scales as N/log(N). More recently, the authors in [17] discussed how to design networks of specific topologies, and how to reach $\alpha_{c}$ values larger than 0.14. Unfortunately, it still finds a bound on the number of limiting behaviours that scale linearly to *N*. Clearly, the optimal storage problem is still open and how to achieve this optimal storage is still the subject of research [18].

The authors in [19] give an analytical expression for the number of retrieval errors, and, when it restricts itself to the region P<N, it retrieves the bound $P<\alpha_{c}N$, which is analytically predicted in [11,12]. On the contrary, when it allows autapses, which implies that the diagonal elements in the connectivity matrix J may be different from zero, it finds that a new region exists, with P≫N, where the number of retrieval errors decreases on increasing *P*. In this new region, the number of retrieval errors reaches values lower than one, thus it finds new favorable conditions for an effective and efficient storage of memory patterns in an RNN. Moreover, it shows that, in this region, the number of storable patterns grows exponentially with *N*. In response to these results, Rocchi et al. [20] found that, in the thermodynamic limit at P≫N, the basin of attraction of the stationary states vanishes. Thus, asymptotically, the basing of attraction of the stationary state coincides with the stationary state itself. Once more, this observation raises a serious doubt on the effective potential of this method for the development of realistic memory storage applications. In this paper, we discuss how to effectively overcome this limitation.

Here, we show that we can overcome the limitations of previous storage approaches when we introduce the concept of “memory neighborhood”, where a neighbor is a state that has a number of flipped neural states smaller than a value *k*. This neighborhood forms a set of redundant network states that are almost identical. Thus, to store a certain memory ξ, we do not use Hebbs’ rule to only store the state ξ, but we apply Hebbs’ rule to the entire neighborhood. We find from the analytical results and the simulations that, because these neighbor states are similar, they end up cooperating to form a set of stable states inside the neighborhood of ξ. Each attractor from this set forms a basin of attraction, and, altogether, the union of their basins of attraction forms a composite basin of attraction that forces the states in the neighborhood to stay inside. We find through analytical considerations that the optimal size of the neighborhoods includes states with neuron patterns that differ at most for 4% of the total neurons. This allows us to store a number of memory patterns that grows exponentially with *N*. Furthermore, surrounding the stored memory patterns, we form a basin of attraction that includes all states with an activation pattern that differs at most for 4% of the neurons.

## 2. How to Use Autapses to Overcome the Hebbian Memory Bound

As we discussed in the introduction, if we use autapses to overcome the P<N bound, the number of attractors increase exponentially, but the attractors’ basins shrink until they contain only a single stationary state. Consequently, to overcome the P<N bound with a Hopfield RNN with autapses, without shrinking the basin of attractions, we propose a strategy based on data storage redundancy. Thus, to generate redundancy, we construct neighborhoods of packed stable states surrounding each memory state that we are trying to store. The states that form this neighborhood are states which are identical to the target memory pattern, except for the activation of a small number of neurons. Once we have a memory and its surrounding neighborhood, we sum together all the corresponding dyadic terms for each neighbor. The reason we do this is that we expect that together these states form a region composed entirely of absorbing states which capture the dynamics of the Hopfield RNN. We find from the analytical discussion and from the experimental validation that, for large *N*, these neighbor states cooperate together absorbing the states in the neighborhood.

Given this attractor storage methodology, we show that the number of patterns with the respective neighborhoods that we are able to store grows exponentially with *N*. Furthermore, we discuss that the number of neighbors needed for an efficient and effective memory storage may be set to grow exponentially with *N*, but at a slower rate than the maximum number of storable memory patterns *P*. This allows for a true advantage in the use of this method. Specifically, we show that this strategy achieves the following three properties: (i) the number of stored memory patterns scales exponentially with *N*; (ii) the basin of attraction size grows exponentially with *N*; and (iii) the stored patterns form a neighborhood region that allows the recognition of the original pattern up to an estimated 4–5% of input errors.

## 3. Discrete Time Hopfield RNN

RNNs’ store information in their connectivity matrix J. For each network, its matrix elements $J_{ij}$ represent the weight of the links between all couples *i* and *j*. If $J_{ij}=0$, the nodes *i* and *j* are disconnected. Otherwise, if $J_{ij}\neq 0$, the nodes *i* and *j* share a link. In this context, $J_{ij}$ quantifies how strongly the neuron *j* affects the neuron *i*. In biological terms, $J_{ij}$ represents how the pre-synaptic signal is transmuted by the dendrite, to be integrated with all the other signals incoming in the cell. More precisely, the $J_{ij}$ modulus indicates the strength of the influence, and the $J_{ij}$ sign indicates the excitatory/inhibitory nature of the interaction. Here, we consider a particularly simple discrete-time RNN with McCulloch–Pitts neurons, and a sign function as the activation function. Given these assumptions, this RNN becomes a Hopfield RNN, and follows the dynamics equation:

(1) $$\sigma_{i}\left(t+1\right)=\theta \left(\sum_{j=1}^{N}J_{ij}\sigma_{j}\left(t\right)\right),$$

where θ(x) is a sign function or Heaviside step function (θ(x)=1 for x≥0, and θ(x)=−1, otherwise), and $\sigma_{i}\left(t\right)\in \left\{-1,1\right\}$. In general, connectivity matrices may be symmetric if for all *i* and *j*, $J_{ij}=J_{ij}$, or asymmetric if there exist an *i* and *j* couple such that $J_{ij}\neq J_{ij}$. A Hopfield RNN allows for both symmetric and asymmetric connectivity matrices.

## 4. Storing Patterns

Research on RNN memory storage was influenced by the pioneering observations discussed by Hebb [21], which lead to the Hebbian rule, and the “Hebbian” approach for memory storage. This approach emphasizes the role of “synaptic plasticity”, and is based on the concept that a neural network is born without connections, such that, for all *i* and *j*, $J_{ij}=0$. Each time, a new memory is stored.

New links are reinforced or added to this network. Thus, the neural network connectivity matrix dynamically evolves as new memories are added. More specifically, in the “Hebbian” approach, to store a set of *P* memory patterns ${\bar{\xi }}^{\left(\mu \right)}$, where μ is an integer index, 1≤μ≤P, we recursively add all the dyadic products of each memory ${\bar{\xi }}^{\left(\mu \right)}$ with itself, and we obtain a connectivity matrix given by

(2) $$J_{ij}=\sum_{\mu =1}^{P}\xi_{i}^{\left(\mu \right)}\xi_{j}^{\left(\mu \right)}.$$

As mentioned earlier, this leaning strategy has an upper limit for the number of patterns *P* that it is able to store [11,12]. The number *P* of memory elements that can be stored must be lower than $\alpha_{c}N$, with $\alpha_{C}\approx 0.14$.

**Figure 1:**
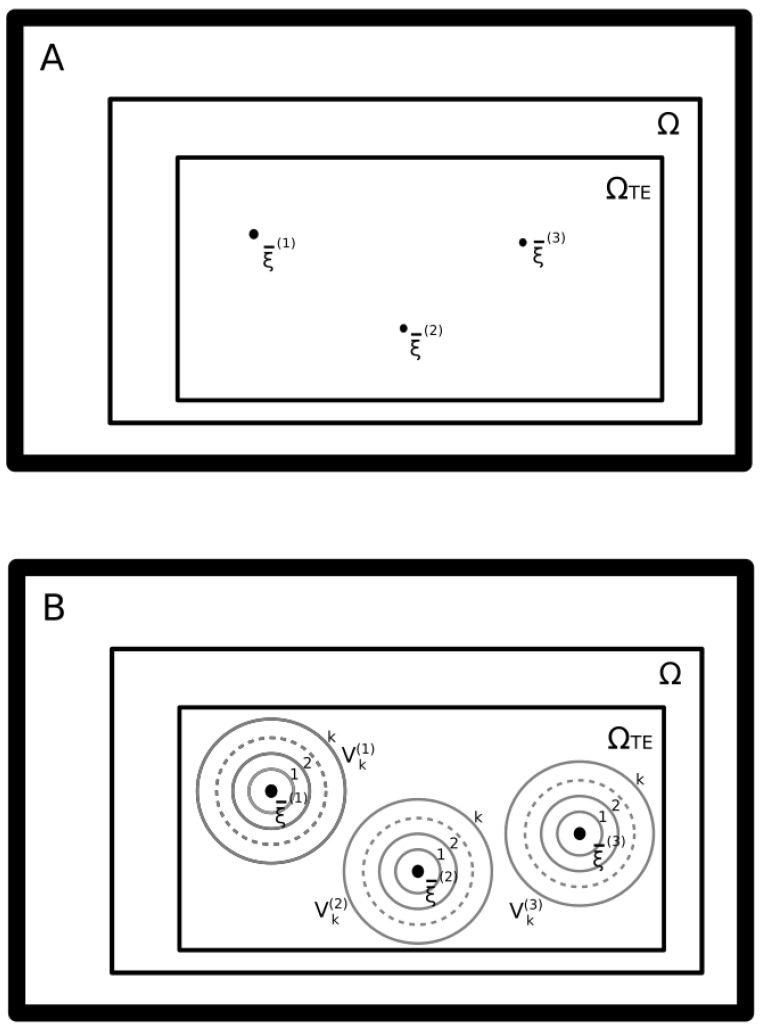
Sketch of the vector states space of a Hopfield RNN. Each point of Ω is one of all the $2^{N}$ possible binary vectors. Among these states, $2^{\gamma N}$ with γ=0.29 may be steady states and belong to $\Omega_{TE}$. (**A**) In the standard approach to memory storage, each stable state is a single memory state, or a spurious memory ${\bar{\xi }}^{\left(\mu \right)}\in \Omega_{TE}$. (**B**) In the approach proposed here, each memory element is represented by a neighborhood of vectors ${\bar{\xi }}^{\left(\mu ,l\right)}\in \Omega_{TE}$ which surrounds the “seed” vector ${\bar{\xi }}^{\left(\mu \right)}$. This neighborhood is obtained by the collection of all vectors ${\bar{\xi }}^{\left(\nu \right)}$ that differ from ${\bar{\xi }}^{\left(\mu \right)}$ of *k*-bits at the most. The neighborhood of ${\bar{\xi }}^{\left(\mu \right)}$ is defined with the shorthand $V_{k}^{\left(\mu \right)}=\left\{{\bar{\xi }}^{\left(\nu \right)}\in \Omega |d\left({\bar{\xi }}^{\left(\mu \right)},{\bar{\xi }}^{\left(\nu \right)}\right)<k\right\}$.

Figure 1 depicts the learning strategy we propose. This strategy is aimed at overcoming the $\alpha_{c}N$ limit. A given memory vector ${\bar{\xi }}^{\left(\mu \right)}$ is represented in the picture as a single point in the *N*-dimensional binary space $\Omega ={\left[0,1\right]}^{N}$. Consequently, Ω collects all possible RNN states, $\left|\Omega \right|=2^{N}$. In general, as discussed earlier, a memory is effectively stored, if it becomes a stable state, regardless of the learning strategy we employ. Thus, given that the recall error is given by the fraction of memory patterns that are not stable states, as more memories are effectively stored, the recall error tends to zero. In the “Hebbian” learning approach, if *P* different memory patterns are stored, then the connectivity matrix J takes the form reported in Equation (2). Figure 1 depicts two conditions that overcome the “Hebbian” learning approach. In the upper pannel, as reported in [19], we show how, given autopses, the number of stored memories *P* can be much larger than *N*. Furthermore, at the same time, autapses allow a retrieval probability larger than the error probability by a factor of *e* in [19]. It is important to point out that, even if greater than *N*, the number of stored patterns *P* must be smaller than $2^{N}$. It is also important to consider that, most likely, *P* must be smaller than the number of average fixed points that are present in a random symmetric fully connected matrix, which is $\Omega_{TE}=2^{\gamma N}$ with γ=0.29 [13]. We thus bound the maximum number of storable points with $\Omega_{TE}$ [13]. Unfortunately, as pointed out by Rocchi et al. [20], the basin of attraction of these points shrinks to the point that it contains only the stable states. In the lower panel, we show how, with our approach, we overcome this limit. Our approach consists of associating a set of neighbor vectors to each memory vector ${\bar{\xi }}^{\left(\mu \right)}$ in order to form a cloud of surrounding neighborhood vectors similar to the original memory. To do this, we have to choose a distance relation in order to define a neighborhood surrounding an arbitrary vector. The distance relation that we choose is the Hamming distance, which, given two binary vectors $\bar{a}$ and $\bar{b}$, is defined as the number of different elements such that $a_{i}\neq b_{j}$. Furthermore, we consider two binary vectors $\bar{a}$ and $\bar{b}$ as neighbors, if they have a Hamming distance d(a,b) smaller than an integer value *k*. Thus, the set of ${\bar{\xi }}^{\left(\mu \right)}$ neighbor vectors is $V_{k}^{\left(\mu \right)}=\left\{{\bar{\xi }}^{\left(\nu \right)}\in \Omega |d\left({\bar{\xi }}^{\left(\mu \right)},{\bar{\xi }}^{\left(\nu \right)}\right)<k\right\}$, where *k* is an integer parameter (k≪N). Given $V_{k}^{\left(\mu \right)}$, the number of vector states that are at a Hamming distance of *k* or lower is $v_{N}\left(k\right)$. Clearly, $v_{N}\left(k\right)$ is equal to the number of elements in $V_{k}^{\left(\mu \right)}$. Consequently, to add the redundancy of all the neighbor states and with the objective of obtaining a neighborhood of attractor stable states, we change the Hebbian rule expressed with Equation (2) with the new rule

(3) $$J_{ij}=\sum_{\mu =1}^{P}\;\;\sum_{{\bar{\xi }}^{\left(\nu \right)}\in V_{k}^{\left(\mu \right)}}\xi_{i}^{\left(\nu \right)}\xi_{j}^{\left(\nu \right)}.$$

In this sum, $Pv_{N}\left(k\right)$ terms are added to form a connectivity matrix J that we expect should store *P* memory states, by forming around each memory state a neighborhood of absorbing states of size $v_{N}\left(k\right)$. Here, we must redefine the concept of recall error because we use a set of absorbing states instead of single absorbing states. Thus, we define the recall error for a neighborhood $V_{k}^{\left(\mu \right)}$, as a failure to retrieve an attractor inside $V_{k}^{\left(\mu \right)}$ starting from a state inside of the neighborhood $V_{k}^{\left(\mu \right)}$. Consequently, the recall error rate would correspond to the rate of states in $V_{k}^{\left(\mu \right)}$ that evolve to attractors outside this state. Now, we have to set *k* considering two constraints, *k* must scale with *N*, and the absorbing-neighborhood must not vanish in the thermodynamic limit. Consequently, we chose β=k/N. This straightforward storage strategy works, if and only if, for each memory pattern, there is enough “room” for all $v_{N}\left(k\right)$ neighbor vector-sets. At the same time, we assume that the redundant stable states we add here should not exceed the theoretical maximum storage capacity that we obtain in a randomly fully connected network. Thus, to fulfill an efficient and effective memory storage, $Pv_{N}\left(k\right)\ll \Omega_{TE}$. The explicit expression for $v_{N}\left(k\right)$ is given by

(4) vN(k)=∑m=0kNm.

Unfortunately, this quantity does not have a closed expression, nevertheless, for k<N/2, we can write a lower and upper bound of the form [22]

(5) $$\frac{2^{NH(\beta )}}{\sqrt{8N\beta (1-\beta )}}<v_{N}\left(K\right)<2^{NH(\beta )},$$

where H(β) is the binary entropy of β, $H\left(\beta \right)=-\beta \log_{2}\left(\beta \right)-\left(1-\beta \right)\log_{2}\left(1-\beta \right)$. If we set aside the logarithmic corrections which vanish in the limit for large *N*, the condition $Pv_{N}\left(k\right)\ll \Omega_{TE}$ can be promptly rewritten as $P\ll P_{c}$ where $P_{c}$ is expressed as

(6) $$P_{c}=2^{N[\gamma -H(\beta )]},$$

with γ=0.29 [13]. This condition tells us that an exponentially large number of memory patterns may be allowed providing that γ>H(β). Clearly, this condition implies $\beta <\beta_{c}=0.051$. Furthermore, if we set

(7) $$\Delta J_{ij}^{\left(\mu \right)}=\sum_{{\bar{\xi }}^{\left(\nu \right)}\in V_{k}^{\left(\mu \right)}}\xi_{i}^{\left(\nu \right)}\xi_{j}^{\left(\nu \right)},$$

then the $\Delta J_{ij}^{\left(\mu \right)}$ are the sums of the contribution of all the ${\bar{\xi }}^{\left(\mu \right)}$’s neighbor vectors. Now, we may rewrite Equation (3) as

(8) $$J_{ij}=\sum_{\mu =1}^{P}\;\Delta J_{ij}^{\left(\mu \right)}.$$

Counting the contribution of the different states that form the neighborhood, Equation (8) can be explicitly expanded.

(9) ΔJij(μ)=∑m=0kNm,ifi=j,∑m=0k[Nm−2N−1m−1−2N−2m−1+2N−2m−2],ifi≠j,ξi(μ)=ξj(μ),∑m=0k[−Nm+2N−1m−1+2N−2m−1−2N−2m−2],otherwise.

Notice that neurons cooperate by either increasing their reciprocal tendency to align or to misalign with each other.

## 5. Simulation Results

To run the simulations, we considered different values of *N*, and selected the values of β and *P* according to Equation (6), because for $P\leq P_{c}$, we expect from the arguments in the previous section that we have a retrieval error close to 0. Indeed, as mentioned previously, β must be smaller than $\beta_{c}=0.051$, thus we chose β=4% because, for β=5%, the value of $P_{c}$ grows extremely slowly and we can take advantage of the exponential scaling of *P* only for large values of *N*. Thus, as a representative case, we show simulations for $P=P_{c}$ with β=4%. Consequently, we run our simulation in the conditions for which we expect that most of the states in the neighborhood $V_{k}^{\left(\mu \right)}$ are recalled, which means that any state in the neighborhood evolves to an attractor in the neighborhood.

**Figure 2:**
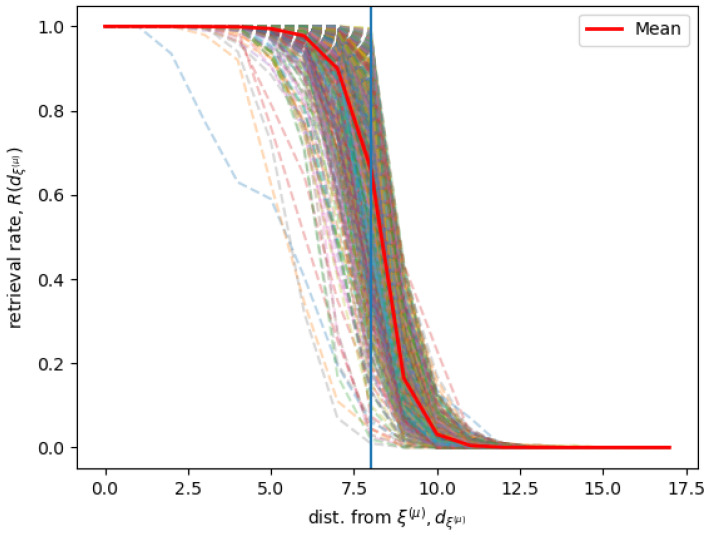
Given the neighborhood $V_{k}^{\left(\mu \right)}$ of each memory ${\bar{\xi }}^{\left(\mu \right)}$, the dashed lines show how the retrieval rate $R(d_{{\bar{\xi }}^{\left(\mu \right)}})$ changes for a sample of neighbors at a distance *d* from ${\bar{\xi }}^{\left(\mu \right)}$. The red line shows the average of $R(d_{{\bar{\xi }}^{\left(\mu \right)}})$ over all ${\bar{\xi }}^{\left(\mu \right)}$. The vertical line represents the β=4% limit which corresponds to the neighborhood bound k=8.

Figure 2 shows simulation results for a Hopfield RNN with a connectivity matrix J trained on 745 memories ${\bar{\xi }}^{\left(\mu \right)}$ with Equation (9). We used n=200 and β=4%, and consequently k=8 and $P=P_{c}=745$. We define the distance $d_{{\bar{\xi }}^{\left(\mu \right)}}$ as the distance of any state from a given memory ${\bar{\xi }}^{\left(\mu \right)}$, and the retrieval rate $R(d_{{\bar{\xi }}^{\left(\mu \right)}})$ at a distance $d_{{\bar{\xi }}^{\left(\mu \right)}}$ as the rate of states at a certain distance $d_{{\bar{\xi }}^{\left(\mu \right)}}$ form the memory ${\bar{\xi }}^{\left(\mu \right)}$ that in the long time limit is attracted by a stable state in the neighborhood of ${\bar{\xi }}^{\left(\mu \right)}$.

The figure shows the $R(d_{{\bar{\xi }}^{\left(\mu \right)}})$ for all the P=745 different stored memories, and the average ${\langle R\left(d_{{\bar{\xi }}^{\left(\mu \right)}}\right)\rangle }_{{\bar{\xi }}^{\left(\mu \right)}}$ computed over all the different memories ${\bar{\xi }}^{\left(\mu \right)}$. Given a memory ${\bar{\xi }}^{\left(\mu \right)}$ and its neighborhood $V_{k}^{\left(\mu \right)}$, for different values of $d_{{\bar{\xi }}^{\left(\mu \right)}}$, the rate $R(d_{{\bar{\xi }}^{\left(\mu \right)}})$ is when possible computed extensively and otherwise sampled, according to the following conditions. If the shell of states in a neighborhood at a certain distance $d_{{\bar{\xi }}^{\left(\mu \right)}}$ has less then 1000 states, the recall rate $R(d_{{\bar{\xi }}^{\left(\mu \right)}})$ is computed considering all the states in the shell. Otherwise, for shells of states at a distance $d_{{\bar{\xi }}^{\left(\mu \right)}}$ with more than 1000 states, only 200 states are sampled. We find that the system has a sharp transition when the state is at the border of the memories neighborhood. This indicates that the states inside of the neighborhood are correctly recalled, while, on average, the states outside the neighborhood are not associated with the neighborhood’s memory.

**Figure 3:**
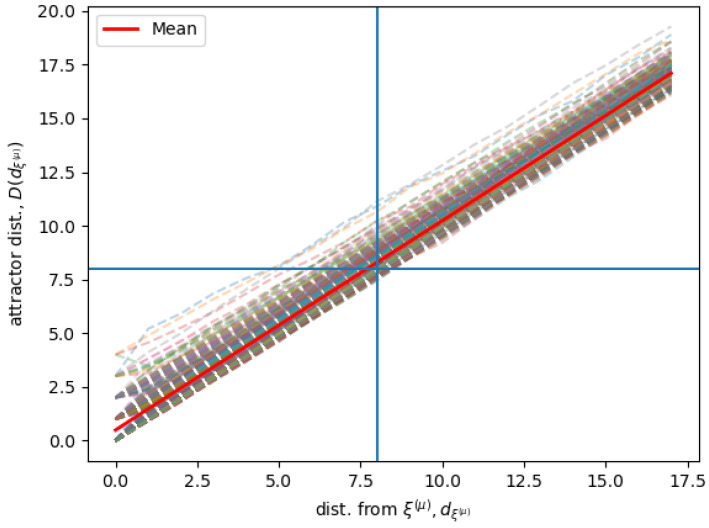
Given each memory ${\bar{\xi }}^{\left(\mu \right)}$ and its neighborhood $V_{k}^{\left(\mu \right)}$, the dashed lines show the average distance from ${\bar{\xi }}^{\left(\mu \right)}$ for the attractors of neighbors at a distance $d_{{\bar{\xi }}^{\left(\mu \right)}}$ from ${\bar{\xi }}^{\left(\mu \right)}$. The red line shows the average of $D(d_{{\bar{\xi }}^{\left(\mu \right)}})$ over all ${\bar{\xi }}^{\left(\mu \right)}$. The vertical and the horizontal line represent the k=8 limit and the neighborhood bound.

Figure 3 shows simulation results for the same Hopfield RNN as for Figure 2, given that each state in the neighborhood is associated with an attractor stable state in the limit behavior. The figure shows the distance of the attractor stable states $D(d_{{\bar{\xi }}^{\left(\mu \right)}})$ for the associated states that are found at a distance $d_{{\bar{\xi }}^{\left(\mu \right)}}$ from each memory ${\bar{\xi }}^{\left(\mu \right)}$. The figure shows $D(d_{{\bar{\xi }}^{\left(\mu \right)}})$ for all the P=745 different stored memories, and the average ${\langle D\left(d_{{\bar{\xi }}^{\left(\mu \right)}}\right)\rangle }_{{\bar{\xi }}^{\left(\mu \right)}}$ computed over all the different memories. $D(d_{{\bar{\xi }}^{\left(\mu \right)}})$ is sampled as in Figure 2.

**Figure 4:**
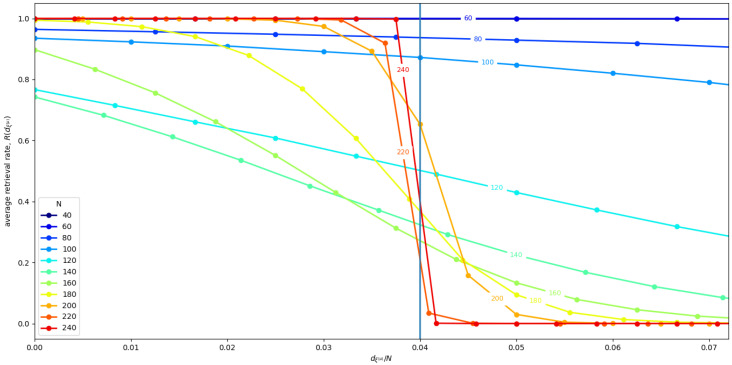
Average ${\langle R\left(d_{{\bar{\xi }}^{\left(\mu \right)}}\right)\rangle }_{r,{\bar{\xi }}^{\left(\mu \right)}}$ computed over eight Hopfield RNN replicas for different *N*, with β=4%, and $P=P_{c}$. The vertical line represents the β=4% limit and the neighborhood bound.

Now, we replicated the simulation eight times for different values of *N*. Each time we selected uniformly at random $P=P_{c}$ solutions, and we generated the corresponding connectivity matrix J with Equation (9), with β=4%. Figure 4 shows the mean ${\langle R\left(d_{{\bar{\xi }}^{\left(\mu \right)}}\right)\rangle }_{r,{\bar{\xi }}^{\left(\mu \right)}}$ averaged over all Hopfield RNN replications *r*, and all the memories ${\bar{\xi }}^{\left(\mu \right)}$ of each replication, for different network sizes *N*. We find that, for N>160, we get the sharp transition behavior that we expected, while, for smaller *N*, we get size effects that influence the stability of the states. We have three different outcomes at different ranges. For small N<80, the size effects do not impact the performance of the storage algorithm, and, on the contrary, seem to increase the basin of attraction of the memories. Differently, in the middle range, e.g., *N*∼100, it appears that the larger number of memories causes them to interfere with each other, completely destroying the basin of attraction of 1 or 2 of the memories. Indeed, in this network, sometimes one or two neighborhoods have a very low retrieval rate. Consequently, the average overall value of ${\langle R\left(d_{{\bar{\xi }}^{\left(\mu \right)}}\right)\rangle }_{r,{\bar{\xi }}^{\left(\mu \right)}}$ decreases.

**Figure 5:**
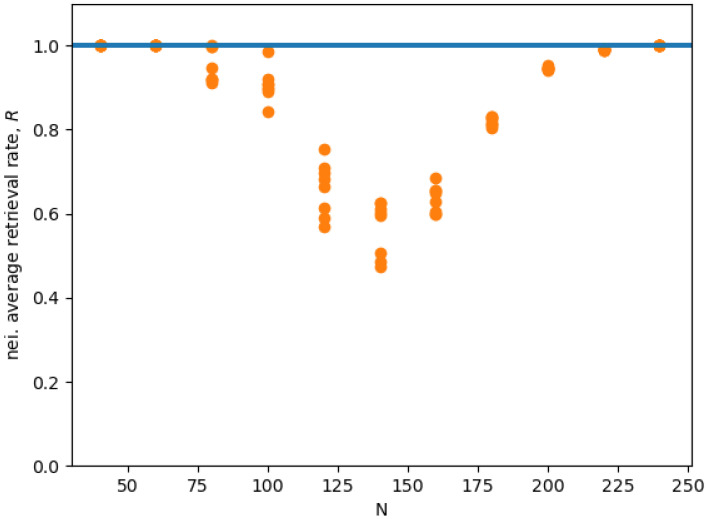
Mean neighborhood retrieval rate *R* for eight Hopfield RNN replicas for different *N* values averaged over all memory states ${\bar{\xi }}^{\left(\mu \right)}$, with β=4%, and $P=P_{c}$. The horizontal line represents the optimal recall of all state in the neighberhood.

Figure 5 shows the average retrieval rate *R* for the state in the neighborhood of each memory ${\bar{\xi }}^{\left(\mu \right)}$. We see that, for small N<80, and, for N>120, we have a retrieval rate close to 1, which indicates an almost optimal retrieval. In the middle range, the retrieval rate is clearly sub-optimal. In conclusion, we speculate that the reason this interference disappears for larger *N* probably has to do with the randomness of the contribution of all the memories’ interacting with any single given memory, as described in [19]. Indeed, in [19], the authors argue that, given a single memory, the effects of the memories interacting with it contribute with a random term. Thus, the total effect of all these random terms coming from different uncorrelated memories vanishes as the number of interacting memories increase as a result of the law of large numbers.

## 6. Conclusions

We have proposed a strategy in which, for each memory vector ${\bar{\xi }}^{\left(\mu \right)}$, we associate a set of neighbor vectors ${\bar{\xi }}^{\left(\nu \right)}\in V_{k}^{\left(\mu \right)}$ defined by a Hamming distance lower than *k*. This strategy allows for each memory to construct a redundant basin of attraction, which is formed by memory patterns with at the most *k* flipped bits. In other words, a stored memory pattern is no longer formed by a single stable state in the binary vector space Ω, but is a cloud of vectors, obtained by surrounding the “seed” memory state with a basin of neighbor vectors, which are formed by all the possible combinations of binary vectors with up to *k* different bits. With this strategy, we demonstrate the three following points: (i) the number of memory patterns *P* that can be stored in a neural network with *N* nodes has an upper bound $P_{c}$, which scales exponentially with *N*, $P_{c}=2^{N[\gamma -H(\beta )]}$; (ii) if the parameter *k* is chosen proportionally to *N*, k=βN, the size of the stored memory neighborhood $v_{N}\left(k\right)$ forms a neighborhood of absorbing vectors for the stored memory which grows exponentially with *N*, $v_{N}\left(k\right)=2^{NH(\beta )}$; (iii) the parameter β has upper bound $\beta_{c}=0.051N$, i.e., the largest basin of attraction for the memory includes patterns which have 5% of errors at the most. We then run simulations to show this results, and we find what we analytically demonstrated. More precisely, we found three ranges. For N>120, we get a sharp transition in the retrieval rate for states inside and outside each memory neighborhood. For 80<N<120, the size effect seriously damages the retrieval rate of each memory neighborhood. For N<80, the size effects have the opposite influence and increase the basin of attraction of the neighborhoods. These findings suggest that Hebbian RNNs with autapses may be used to construct efficient and effective data storage systems that can store a number of solutions that grows exponentially with *N*.

## Abbreviations

The following abbreviations are used in this manuscript:

RNN	Recurrent Neural Network
